# M6A-methylated circPOLR2B forms an R-loop and regulates the biological behavior of glioma stem cells through positive feedback loops

**DOI:** 10.1038/s41419-024-06946-6

**Published:** 2024-08-01

**Authors:** Hongda Lin, Zheng Cui, Tiange E, Hailing Xu, Di Wang, Ping Wang, Xuelei Ruan, Libo Liu, Yixue Xue

**Affiliations:** 1 Department of Neurosurgery, Shengjing Hospital of China Medical University, Shenyang, China; 2 Key Laboratory of Neuro-oncology in Liaoning Province, Shenyang, China; 3 Liaoning Medical Surgery and Rehabilitation Robot Technology Engineering Research Center, Shenyang, China; 4 Department of Neurobiology, School of Life Sciences, China Medical University, Shenyang, 110122 China

**Keywords:** CNS cancer, Cancer stem cells

## Abstract

Glioma is the most common primary brain tumor, and targeting glioma stem cells (GSCs) has become a key aspect of glioma treatment. In this study, we discovered a molecular network in which circRNA forms an R-loop structure with its parental gene to regulate the biological behavior of GSCs. Genes with abnormal expression in GSCs were screened using RNA-seq and circRNA microarray analyses. The study results showed that high expression of YTHDC1 in GSCs promoted the transportation of N6-methyladenosine (m6A)-modified circPOLR2B from the nucleus to the cytoplasm. Decreased circPOLR2B levels in the nucleus resulted in fewer R-loop structures formed with its parental gene POLR2B. This reduction in R-loop structures relieved the inhibitory effect on POLR2B transcription and upregulated PBX1 expression through alternative polyadenylation (APA) action, thereby promoting the malignant biological behavior of GSCs. Knockdown of YTHDC1, POLR2B, and PBX1 reduced xenograft tumor volume and prolonged the survival of nude mice. The YTHDC1/circPOLR2B/POLR2B/PBX1 axis plays a regulatory role in the biological behavior of GSCs, offering potential targets and novel strategies for the treatment of glioma.

## Introduction

Glioblastoma (GBM) is the most malignant primary intracranial tumor, with the highest incidence rate occurring in adults and a median survival period of under 2 years [1, 2]. Cell heterogeneity can lead to GBM invasion and metastasis to the entire brain [3]. Certain cells may self-renew and differentiate in a manner similar to that of normal stem cells, and malignant parenchymal tumors contain their own stem cells, known as cancer stem cells (CSCs) [4]. An increasing number of studies have indicated that CSCs can promote the occurrence, metastasis, and recurrence of tumors and increase their chemotherapy resistance; CSCs are also the primary source of tumor cell heterogeneity [5]. Glioma stem cells (GSCs) can self-renew, invade, and migrate, providing key evidence for the recurrence and formation of new intracranial cancerous lesions after GBM surgery [6]. Therefore, targeting GSCs is likely key to treating GBM.

Previous studies have shown that N6-methyladenosine (m6A) modification promotes the self-renewal of GSCs and is crucial for maintaining their dedifferentiation state, thus promoting the growth of GBM [7, 8]. M6A modification is the most abundant RNA modification in mammals, typically embedded in a common sequence of 5′-RRACH-3′ (R = G/A/U, H = U/A/C) [9]. M6A modifications regulate RNA processing, splicing, degradation, nuclear export, and translation through writers, erasers, and readers, thereby regulating RNA expression and function [10, 11]. The m6A methylation modification of RNA is dynamically regulated through the m6A writer and eraser [12]. Methyltransferase 3 (METTL3), methyltransferase 14 (METTL14), and WT1-associated protein (WTAP) complexes are m6A writers [13]. Meanwhile, Fat Mass and Obesity-Associated Gene (FTO) and alkB homolog 5 (ALKBH5) are m6A demethylases, known as erasers [14, 15]. In addition, m6A-modified RNA needs to be recognized by readers in order to exert its effects [16]. YTH N6-methyladenosine RNA-binding protein C1 (YTHDC1) belongs to the YTH domain protein family. As m6A readers, the proteins in this family contain a conserved YTH domain at the C-terminus, the most crucial domain for binding m6A-modified RNA [17]. Previous studies have shown that YTHDC1 can enhance the transport ability of m6A-modified mRNA to the cytoplasm [18]. YTHDC1 also enhances the export of m6A-modified circHPS5 to the cytoplasm, thereby promoting liver cancer progression [19]. However, the role of YTHDC1 in regulating the nucleocytoplasmic distribution of non-coding RNA (ncRNA) in GSCs is unclear.

Circular RNAs (circRNAs) are a type of highly stable ncRNA with a closed circular structure and no 5′ terminal cap or 3′ terminal poly (A) tail, and are therefore unaffected by RNA exonucleases [20]. Current research shows that circRNAs can participate in regulating the biological behavior of various cancer cells by acting as molecular sponges for microRNAs (miRNAs), interacting with proteins, translating proteins, or regulating gene transcription [21, 22]. In colorectal cancer, circRERE plays the role of a molecular sponge by competitively binding to miR-6837-3p, thereby protecting mitochondrial antiviral signaling protein (MAVS) mRNA from degradation and promoting anti-tumor immunity [23]. Similarly, hsa_circ_0003258 upregulates Rho GTPase activating protein 5 (ARHGAP5) via molecular sponge action to promote prostate cancer metastasis [24]. Hsa_circ_000613 binds to ELAV-like RNA-binding protein 1 (ELAV1, an RNA-binding protein, also known as HuR) to protect ELAV1 from ubiquitination and promotes the development of gastric cancer [25]. CircHECTD1-463aa (amino acid), a peptide encoded by hsa_circ_0002301 and downregulated in glioma, attenuates ubiquitination of Nuclear receptor subfamily 2 group F member 1 (NR2F1) and promotes tumor vasculogenic mimicry [26]. According to reports, m6A-modified circRNA is involved in regulating the development of various cancers such as prostate cancer and hepatocellular carcinoma, but its regulatory process in glioma is unclear [27, 28]. The CircBank and SRAMP databases were used to identify hsa_circ_0126701 (circPOLR2B) with a length of 396 nt and potential m6A methylation sites [29, 30].

Pre-B cell leukemia homeobox 1 (PBX1) is a transcription factor upregulated in various malignant tumors [31, 32]. High PBX1 expression promotes GTP cyclohydrolase 1 (GCH1) transcription and the malignant progression of gastric cancer [33]. PBX1 further upregulates the signal transducer and activator of transcription 3 (STAT3) expression through transcription, thereby enhancing the resistance of ovarian cancer cells to carboplatin [34]. However, the mechanism by which PBX1 regulates the biological behavior of GSCs remains unclear.

In this study, Nestin (NES) and SRY-box transcription factor 2 (SOX2) were identified as downstream factors of PBX1, which are regulated by the transcriptional promotion effect of PBX1. NES and SOX2 are recognized as stemness markers of GSCs [35]. High NES expression is positively correlated with poor prognosis in patients with GBM, and GSCs exhibiting high levels of NES expression demonstrate stronger malignant biological behavior [36]. Meanwhile, high SOX2 expression is a key factor driving the tumorigenesis of GSCs, playing a crucial role in maintaining cellular stemness and promoting cell invasion [37, 38]. Therefore, targeting NES and SOX2 to inhibit the malignant biological behavior of GSCs has become crucial in GBM treatment.

## Results

### High expression of YTHDC1 promotes the malignant biological behavior of GSCs

**Fig. 1:**
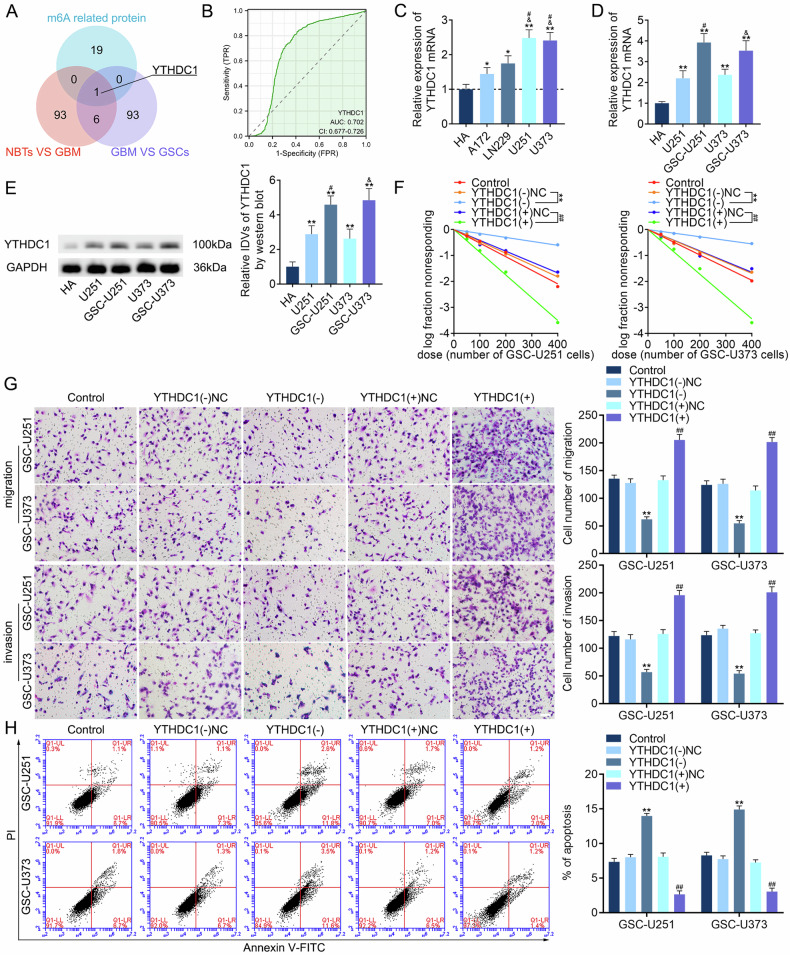
High YTHDC1 expression promotes the malignant biological behavior of GSCs. **A** Using the GTEx project, TCGA database, and RNA-seq screening, upregulated genes were identified and combined with m6A-related genes to screen YTHDC1. **B** ROC curve of YTHDC1 gene expression in the GBM queue of TCGA. **C** YTHDC1 expression in GBM cell lines differs from that in HA. Data are presented as mean ± standard deviation (SD; *n* = 3, each group). *P < 0.05 compared with the HA group, **P < 0.01 compared with the HA group, ^#^*P* < 0.05 compared with the A172 group, and ^&^*P* < 0.05 compared with the LN229 group. **D**, **E** The expression of YTHDC1 in HA, U251, GSC-U251, U373, and GSC-U373, and the statistical analysis of integrated densities (IDVs) in each group. Data are presented as mean ± SD (*n* = 3, each group). **P < 0.01 compared with the HA group, ^#^*P* < 0.05 compared with the U251 group, and ^&^*P* < 0.05 compared with the U373 group. **F** ELDA was used to detect the impact of the knockdown or overexpression of YTHDC1 on the self-renewal ability of GSCs. **P < 0.01 compared with the YTHDC1(−)NC group and ^##^*P* < 0.01 compared with the YTHDC1(+)NC group. **G** The Transwell assay was used to detect the impact of the knockdown or overexpression of YTHDC1 on the migration and invasion ability of GSCs. Data are presented as mean ± SD (*n* = 3, each group). **P < 0.01 compared with the YTHDC1(−)NC group and ^##^*P* < 0.01 compared with the YTHDC1(+)NC group. **H** Flow cytometry was used to detect the impact of the knockdown or overexpression of YTHDC1 on the apoptosis level of GSCs. Data are presented as mean ± SD (*n* = 3, each group). **P < 0.01 compared with the YTHDC1(−)NC group and ^##^*P* < 0.01 compared with the YTHDC1(+)NC group.

The Genotype-Tissue Expression (GTEx) project and The Cancer Genome Atlas (TCGA) database were used to screen for the top 100 genes significantly upregulated in GBM tissues compared to those in normal brain tissues (NBTs), according to log2 change multiples (Fig. S1A). RNA-seq was used to screen the top 100 genes with higher expression in GSCs than in GBM cells, which may be related to the maintenance of the stemness of GSCs and the promotion of the malignant biological behavior of GSCs (Fig. S1B). Because m6A methylation plays a crucial regulatory role in the self-renewal and tumorigenesis of GSCs [8, 39], YTHDC1 was screened after selecting 20 m6A-related genes (Fig. 1A, Fig. S1C, Supplementary Table 1). A receiver operating characteristic (ROC) curve was drawn to determine the diagnostic value of YTHDC1 in GBM; the area under the curve (AUC) was 0.702 (Fig. 1B). The Cancer Cell Line Encyclopedia (CCLE) database was used to obtain an expression matrix of YTHDC1 in various GBM cell lines [40] (Fig. S1D). Finally, the U251 and U373 cell lines were selected (Fig. 1C). Compared to that in normal human astrocytes (HA), YTHDC1 expression was upregulated in GBM cells and significantly elevated in GSCs (Fig. 1D, E). Using immunofluorescence (IF), the YTHDC1 protein was found to be localized in the nucleus (Fig. S1E). We validated the self-renewal ability of GSCs using an extreme limiting dilution analysis (ELDA) experiment and compared it to that of the negative control (NC) group. We also validated the changes in GSC migration and invasion ability through Transwell experiments, and identified apoptotic cells using flow cytometry and the Annexin V-FITC/propidium iodide (PI) combined staining method. Knockdown of YTHDC1 expression inhibited the self-renewal, migration, and invasion of GSCs and promoted apoptosis (Fig. 1F–H). YTHDC1 knockdown further led to significantly decreased NES and SOX2 expression (Fig. S1F), whereas YTHDC1 overexpression significantly reversed these effects.

### CircPOLR2B is downregulated in the nuclei of GSCs and regulates their biological behavior

**Fig. 2:**
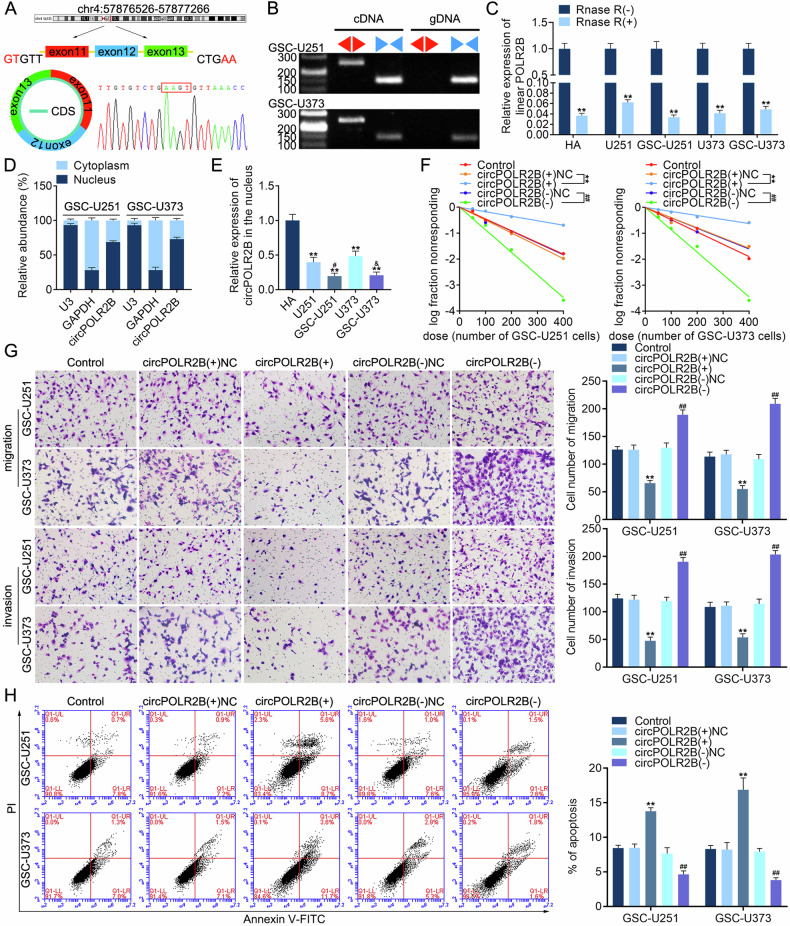
CircPOLR2B is downregulated in the nuclei of GSCs and regulates their biological behavior. **A** CircPOLR2B derived from POLR2B pre-mRNA exons 11–13 and confirmed by Sanger sequencing across the junction region. **B** PCR with diverging (red) and converging primers (blue) were used to detect circPOLR2B in cDNA and gDNA. **C** The qRT-PCR assay was used to detect the digestion efficiency of RNase R. Data are presented as mean ± SD (*n* = 3, each group). **P < 0.01 compared with the RNase R(−) group. **D** The qRT-PCR assay determined that circPOLR2B is codistributed in the nucleus and cytoplasm and is more concentrated in the nucleus. GAPDH and U3 served as internal references for the cytoplasm and nucleus. **E** Expression of circPOLR2B in HA, U251, GSC-U251, U373, and GSC-U373. Data are presented as mean ± SD (*n* = 3, each group). **P < 0.01 compared with the HA group, ^#^*P* < 0.05 compared with the U251 group, and ^&^*P* < 0.05 compared with the U373 group. Effects of circPOLR2B on the self-renewal (**F**), migration and invasion (**G**), and apoptosis levels (**H**) of GSC-U251 and GSC-U373. **P < 0.01 compared with the circPOLR2B(+)NC group and ^##^*P* < 0.01 compared with the circPOLR2B(−)NC group. The scale bar represents 10 μm.

RNA was extracted from the nuclei of GSCs and analyzed using a microarray analysis to detect changes in circRNA expression after YTHDC1 overexpression. Reportedly, in hepatocellular carcinoma (HCC), YTHDC1 helps m6A-modified circHPS5 to exit the nucleus, increasing the number of HCC stem cell spheres and upregulating the expression of stem cell marker CD133 [19]. Therefore, we also chose to downregulate the expression of circRNAs in the nucleus after overexpressing YTHDC1, with has_circ_0126701 (circPOLR2B) being the most markedly downregulated (Fig. S2A, B). After searching the CircBank database (www.circbank.cn), we found that circPOLR2B comprises RNA polymerase II subunit B (POLR2B) exons 11–13. According to the characteristics of its 3′ and 5′ ends, the divergent and convergent primers of the cross-loop junction site were designed and the sequence of the cross-loop junction site was obtained, which proved that circPOLR2B did not exist in gDNA (Fig. 2A, B). Furthermore, an RNase R assay was used to analyze linear RNA digestion efficiency (Fig. 2C). The actinomycin D assay showed that circPOLR2B was more stable than the POLR2B mRNA (Fig. S2C). We also found that circPOLR2B showed nucleocytoplasmic codistribution and was primarily concentrated in the nucleus (Fig. 2D). Therefore, we extracted nuclear RNA and found that compared to that in HA, circPOLR2B expression was downregulated in the nucleus of GBM cells and was more distinct in GSCs (Fig. 2E). CircPOLR2B overexpression inhibited the self-renewal, migration, and invasion abilities of GSCs and promoted apoptosis (Fig. 2F–H), further inhibiting the expression of NES and SOX2 (Fig. S2D), whereas circPOLR2B knockdown reversed these effects.

### YTHDC1 regulates the nucleocytoplasmic distribution of circPOLR2B through m6A methylation

**Fig. 3:**
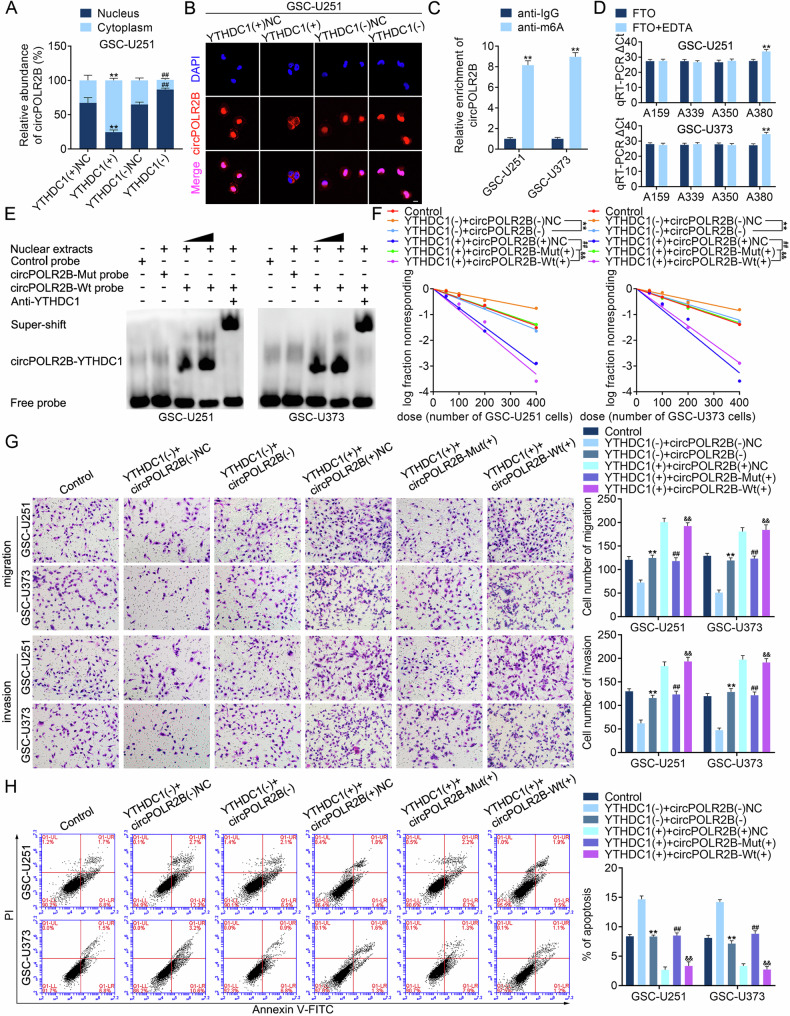
YTHDC1 regulates the nuclear–cytoplasmic distribution of circPOLR2B through m6A methylation modifications. **A**, **B** qRT-PCR and FISH assays showed that after the overexpression of YTHDC1 in GSC-U251, circPOLR2B in the nucleus transferred to the cytoplasm, while knockdown of YTHDC1 resulted in circPOLR2B remaining in the nucleus (the scale bar represents 10 μm). Data are presented as mean ± SD (*n* = 3, each group). **P < 0.01 compared with the YTHDC1(+)NC group, and ^##^*P* < 0.01 compared with the YTHDC1(−)NC group. **C** The MeRIP assay showed that circPOLR2B was enriched. Data are presented as mean ± SD (*n* = 3, each group). **P < 0.01 compared with the anti-immunoglobulin G (IgG) group. **D** qRT-PCR showed difficulty in amplifying circPOLR2B at A_380_. Data are presented as mean ± SD (*n* = 3, each group). **P < 0.01 compared with the FTO group. **E** The EMSA assay confirmed the binding of YTHDC1 to the circPOLR2B A_380_ site. Effects of YTHDC1 and circPOLR2B-Mut or circPOLR2B-Wt on the self-renewal (**F**), migration and invasion (**G**), and apoptosis levels (**H**) of GSC-U251 and GSC-U373. **P < 0.01 compared with the YTHDC1(−) + circPOLR2B(−)NC group, ^##^*P* < 0.01 compared with the YTHDC1(+) + circPOLR2B(+)NC group, and ^&&^*P* < 0.01 compared with the YTHDC1(+) + circPOLR2B-Mut(+) group. The scale bar represents 10 μm.

qRT-PCR and fluorescence in situ hybridization (FISH) assays revealed that YTHDC1 overexpression promoted the transfer of circPOLR2B from the nucleus to the cytoplasm, and knockdown of YTHDC1 caused circPOLR2B to remain in the nucleus of GSC-U251 (Fig. 3A, B) and GSC-U373 (Fig. S3A–B). A search on the SRAMP website (www.cuilab.cn) revealed the presence of four potential m6A methylation sites (A_159_, A_339_, A_350_, and A_380_) in circPOLR2B (Fig. S3C). A methylated RNA immunoprecipitation (MeRIP) assay targeting m6A modifications revealed that circPOLR2B was enriched (Fig. 3C). In this study, we used a particular DNA ligase that could hinder the PCR extension process at m6A methylation sites. The m6A methylation of circPOLR2B could be eliminated by the m6A eraser FTO. However, m6A methylation could not be eliminated by the addition of a denatured FTO (FTO + EDTA) group. The qRT-PCR assay revealed that the cycle threshold (ΔCt) between the FTO and inactive FTO (FTO + EDTA) groups was different at the A_380_ site (Fig. 3D). This was attributed to m6A methylation at the A_380_ site in the FTO + EDTA group, which hindered qRT-PCR progress [41, 42]. A mutated circPOLR2B (circPOLR2B mutant type, circPOLR2B-Mut) probe for m6A methylation at the A_380_ site was designed (Fig. S3D). The electrophoretic mobility shift assay (EMSA) demonstrated that YTHDC1 binds circPOLR2B through the A_380_ site (Fig. 3E), and molecular docking was performed using ZDOCK software [43] (Fig. S3E). To further confirm that the regulation of circPOLR2B by YTHDC1 is mediated by m6A methylation, dot blot assays were performed and revealed that, compared with that in HA, the m6A methylation level of circRNAs in GBM cells increased and the upregulation was more distinct in GSCs, indicating that m6A modification of circRNAs may widely exist in GBM cells and GSCs (Fig. S3F). Recent studies have shown that METTL3, METTL14, and WTAP often form trimers to jointly exert catalytic effects, among which METTL3 has been identified as the catalytic core of the methyltransferase complex, directly participating in the modification of m6A [44, 45]. We found in our experiment that after the knockdown of METTL3, the m6A methylation level of circRNAs decreased (Fig. S3G). A qRT-PCR assay showed that the knockdown of METTL3 expression in GSCs increased the proportion of circPOLR2B in the nucleus; that is, the ability of circPOLR2B to exit the nucleus was weakened (Fig. S3H). These results suggest that YTHDC1 regulates the nucleocytoplasmic distribution of circPOLR2B through the circPOLR2B A_380_ m6A modification site.

Additionally, rescue assays were performed to elucidate the regulatory effects of YTHDC1 and circPOLR2B on GSCs. The results showed that circPOLR2B knockdown after YTHDC1 knockdown reversed the inhibitory effect of YTHDC1 knockdown on the malignant biological behavior of GSCs, as well as the inhibitory effect of YTHDC1 knockdown on the expression of NES and SOX2 (Fig. 3F–H, Fig. S3I). However, YTHDC1 overexpression followed by circPOLR2B-Mut overexpression reversed the promoting effect of YTHDC1 overexpression on the malignant biological behavior of GSCs, as well as the promoting effect of YTHDC1 overexpression on the expression of NES and SOX2 (Fig. 3F–H, Fig. S3I). Subsequently, we designed a circPOLR2B overexpression vector that can undergo m6A methylation at the A_380_ site, which can forcibly express circPOLR2B modified with m6A at the A_380_ site, and named it circPOLR2B wild type (circPOLR2B-Wt). Notably, the overexpression of circPOLR2B-Mut (rather than circPOLR2B-Wt) after YTHDC1 overexpression can reverse the promoting effect of YTHDC1 overexpression on the malignant biological behavior of GSCs and the expression promotion of NES and SOX2. However, compared with the group that only overexpressed YTHDC1, circPOLR2B-Wt overexpression on the basis of YTHDC1 overexpression did not alter the self-renewal, migration, and invasion ability of GSCs, nor their apoptosis level. The expression levels of NES and SOX2 did not change either (Fig. 3F–H, Fig. S3I). YTHDC1 mediates the nuclear efflux effect of circPOLR2B-Wt to counteract the effect of circPOLR2B-Wt overexpression in the nucleus, while circPOLR2B-Mut is not recognized by YTHDC1 owing to the m6A methylation site mutation of A_380_, indicating a lack of nuclear output ability. Overall, our rescue experiments have demonstrated that circPOLR2B is a downstream factor of YTHDC1, and YTHDC1 regulates the biological behavior of GSCs by regulating the nuclear–cytoplasmic distribution of circPOLR2B through m6A methylation.

### CircPOLR2B forms an R-loop structure with the parental gene POLR2B

**Fig. 4:**
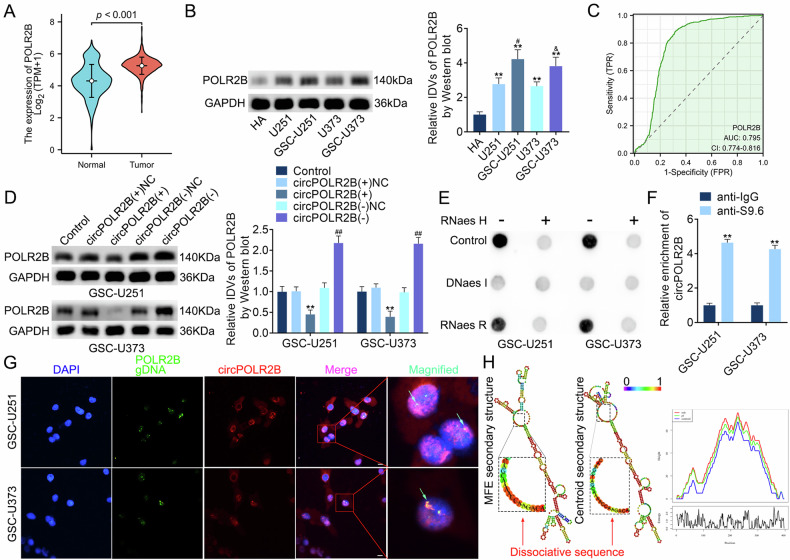
CircPOLR2B forms an R-loop structure with the parent gene POLR2B. **A** POLR2B expression is higher in tumor tissues than in normal tissues. **B** Expression of POLR2B in HA, U251, GSC-U251, U373, and GSC-U373; the IDVs in each group were statistically analyzed. Data are presented as mean ± SD (*n* = 3, each group). **P < 0.01 compared with the HA group, ^#^*P* < 0.05 compared with the U251 group, and ^&^*P* < 0.05 compared with the U373 group. **C** ROC curve of POLR2B expression in TCGA–GBM queue. **D** The effects of the overexpression or knockdown of circPOLR2B on POLR2B expression; the IDVs in each group were statistically analyzed. Data are presented as mean ± SD (*n* = 3, each group). **P < 0.01 compared with the circPOLR2B(+)NC group and ^##^*P* < 0.01 compared with the circPOLR2B(−)NC group. **E** After treatment with RNase H, DNase I, or RNase R, the S9.6 dot blot assay was performed. **F** CircPOLR2B was enriched in the DRIP assay. Data are presented as mean ± SD (*n* = 3, each group). **P < 0.01 compared with the anti-IgG group. **G** CircPOLR2B combined with its parent gene POLR2B gDNA to form an R-loop structure. The nucleus was stained with DAPI (scale, 10 μm). **H** The RNAFOLD web server was used to predict the secondary structure of circPOLR2B.

Upon determining the mechanism by which only the expression level of circPOLR2B in the nucleus regulates the biological behavior of GSCs, we analyzed data from the GTEx project and TCGA database and found that POLR2B expression is higher in glioma tissues than in normal tissues (Fig. 4A) and that compared to that in HA, the expression of POLR2B was upregulated in GBM cells and was more pronounced in GSCs (Fig. 4B). The diagnostic value of POLR2B was then evaluated using a ROC curve with an AUC of 0.795 (Fig. 4C). POLR2B knockdown inhibited the expression of NES and SOX2 in GSCs. The self-renewal, migration, and invasion abilities of the cells were weakened, and cell apoptosis was accelerated. Meanwhile, POLR2B overexpression exerted the opposite effects (Fig. S4A–D). We also found that circPOLR2B overexpression inhibited the expression of POLR2B, whereas circPOLR2B knockdown upregulated the expression of POLR2B (Fig. 4D). An S9.6 dot blot assay revealed that the R-loop structure, composed of circRNAs, may widely exist in GSCs (Fig. 4E). The DRIP assay revealed that circPOLR2B was involved in the formation of R-loops in GSCs (Fig. 4F). In DNA–RNA FISH, circPOLR2B bound to its parental gene, POLR2B gDNA (Fig. 4G). To discover potential binding sites for circPOLR2B to form the R-loop structure, we accessed the RNAFOLD website (http://rna.tbi.univie.ac.at/) to search for the secondary structure of circPOLR2B [46] (Fig. 4H). A potential binding site, 5′-UAGCAAAACCAAGACA-3′, was found on circPOLR2B (Fig. 4H), and the site was designed to be mutated into a circPOLR2B binding-defective type (circPOLR2B-BD; Figure S4E). To confirm this binding site, we overexpressed circPOLR2B-BD after circPOLR2B knockdown, and the DRIP assay indicated that circPOLR2B-BD was not enriched and did not participate in R-loop formation (Figure S4F). These results demonstrated that circPOLR2B was combined with the parental gene POLR2B through the 5′-UAGCAAAACCAAGACA-3′ site, forming an R-loop structure.

### CircPOLR2B regulates POLR2B expression at exon 15

**Fig. 5:**
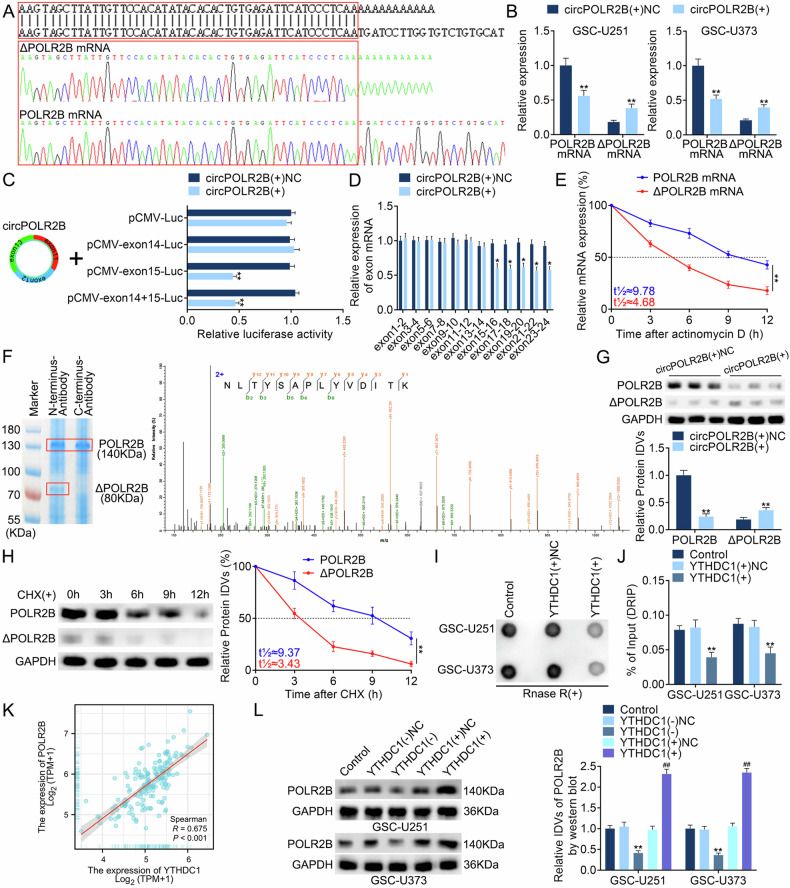
CircPOLR2B regulates POLR2B expression at exon 15. **A** Sanger sequencing confirmed that circPOLR2B is truncated at exon 15 of the POLR2B gene and generates ΔPOLR2B mRNA. **B** After overexpressing circPOLR2B, ΔPOLR2B mRNA expression is upregulated, while normal POLR2B mRNA expression is downregulated. Data are presented as mean ± SD (*n* = 3, each group). **P < 0.01 compared with the circPOLR2B(+)NC group. **C** The overexpression of circPOLR2B in 293T cells resulted in the decreased fluorescence activity of the construction vector containing the exon 15 sequence. Data are presented as mean ± SD (*n* = 3, each group). **P < 0.01 compared with the circPOLR2B(+)NC group. **D** After the overexpression of circPOLR2B, the expression of exon 15 and subsequent exons of POLR2B was downregulated. Data are presented as mean ± SD (*n* = 3, each group). **P < 0.01 compared with the circPOLR2B(+)NC group. **E** After actinomycin D treatment, the relationship between the half-lives of POLR2B mRNA and ΔPOLR2B mRNA was analyzed. Data are presented as mean ± SD (*n* = 3, each group). **P < 0.01 compared with the POLR2B mRNA group. **F** An additional protein was pulled down against the POLR2B-N-terminal antibody and confirmed by mass spectrometry to be the truncated ΔPOLR2B protein. **G** ΔPOLR2B expression upregulation and normal POLR2B protein expression downregulation after overexpressing circPOLR2B; the IDVs in each group were statistically analyzed. Data are presented as mean ± SD (*n* = 3, each group). **P < 0.01 compared with the circPOLR2B(+)NC group. **H** Protein half-life after treating cells with CHX; the IDVs in each group were statistically analyzed. Data are presented as mean ± SD (*n* = 3, each group). **P < 0.01 compared with the POLR2B group. **I**, **J** After YTHDC1 overexpression in GSCs, the formation of the R-loop structure by circRNAs was reduced, while the enrichment of circPOLR2B was reduced in DRIP. Data are presented as mean ± SD (*n* = 3 in each group). **P < 0.01 compared with the YTHDC1(+)NC group. **K** TCGA–GBM database showcased the relationship between the expression levels of YTHDC1 and POLR2B. **L** Effects of YTHDC1 on POLR2B expression; the IDVs in each group were statistically analyzed. Data are presented as mean ± SD (*n* = 3, each group). **P < 0.01 compared with the YTHDC1(−)NC group and ^##^*P* < 0.01 compared with the YTHDC1(+)NC group.

To explore the mechanisms underlying the circPOLR2B-mediated regulation of POLR2B expression, this study used the 3′ rapid amplification of cDNA end (RACE) assay to obtain different length sequences in addition to the normal POLR2B mRNA sequence. Sanger sequencing revealed that there was a truncated POLR2B mRNA at exon 15 (ΔPOLR2B mRNA; Fig. 5A). qRT-PCR assays revealed that after the overexpression of circPOLR2B, ΔPOLR2B mRNA expression was elevated, while normal POLR2B mRNA expression was downregulated (Fig. 5B). Luciferase reporter assays found that circPOLR2B bound to POLR2B in exon 15 and prevented the transcription of subsequent sequences (Fig. 5C), which was confirmed by qRT-PCR spanning two exons (Fig. 5D). The actinomycin D assay showed that compared to that of normal POLR2B mRNA, ΔPOLR2B mRNA stability was reduced (Fig. 5E). Subsequently, antibodies against the N- and C-termini of POLR2B were designed. The antibody against the N-terminus, but not the C-terminus, pulled down another unknown protein besides POLR2B. Sequence alignment mass spectrometry revealed that ΔPOLR2B mRNA transcribed the same truncated POLR2B protein (ΔPOLR2B; Fig. 5F). The overexpression of circPOLR2B resulted in ΔPOLR2B expression being elevated and normal POLR2B expression being reduced (Fig. 5G). We also found that the half-life of ΔPOLR2B was shorter than that of the normal POLR2B (Fig. 5H). Furthermore, overexpressed ΔPOLR2B did not change NES or SOX2 expression in GSCs, and the self-renewal efficiency did not change, suggesting that ΔPOLR2B is a nonfunctional protein (Fig. S5A, B). Rescue assays showed that the dual overexpression of circPOLR2B and POLR2B reversed the inhibition of the malignant biological behavior of GSCs by circPOLR2B overexpression and simultaneously reversed the inhibition of NES and SOX2 expression. The double knockdown of circPOLR2B and POLR2B reversed the promoting effect of circPOLR2B knockdown on the malignant biological behavior of GSCs and simultaneously reversed the promoting effect of NES and SOX2 expression (Fig. S5C–F). Finally, the S9.6 dot blot assay revealed that YTHDC1 overexpression reduced the formation of the R-loop structure involved in circRNAs in GSCs, and after overexpressing YTHDC1, the enrichment of circPOLR2B decreased in the DRIP assay, demonstrating the existence of the YTHDC1/circPOLR2B/POLR2B pathway (Fig. 5I, J). Further analysis revealed a positive correlation between the expression levels of YTHDC1 and POLR2B in the TCGA–GBM database (Fig. 5K). We found that overexpression of YTHDC1 in GSC-U251 and GSC-U373 resulted in an increase in POLR2B expression levels. Similarly, knocking down YTHDC1 leads to a downregulation of POLR2B expression levels (Fig. 5L). In summary, we have demonstrated that YTHDC1 can regulate the expression level of POLR2B by altering the nucleocytoplasmic distribution of circPOLR2B.

### POLR2B regulates PBX1 expression through alternative polyadenylation (APA) action

**Fig. 6:**
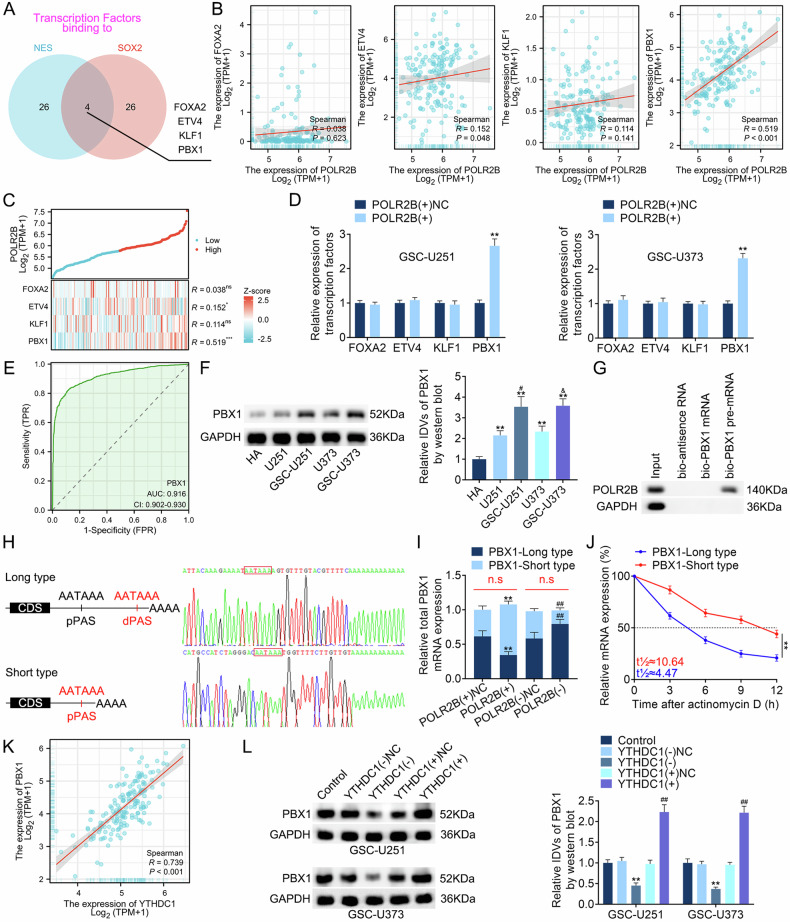
POLR2B regulates PBX1 expression through APA action. **A** Transcription factors that bind to the NES and SOX2 promoter regions were screened in the HumanTFDB. **B** TCGA–GBM database showcased the expression levels of POLR2B and four transcription factors. **C** Co-expression heatmap of POLR2B with FOXA2, ETV4, KLF1, and PBX1. **D** After POLR2B overexpression, PBX1 upregulation was significant. Data are presented as mean ± SD (*n* = 3, each group). **P < 0.01 compared with the POLR2B(+)NC group. **E** ROC curve of PBX1 expression in the GBM queue of TCGA. **F** The expression of PBX1 in HA, U251, GSC-U251, U373, and GSC-U373. Data are presented as mean ± SD (*n* = 3, each group). **P < 0.01 compared with the HA group, ^#^*P* < 0.05 compared with the U251 group, and ^&^*P* < 0.05 compared with the U373 group. **G** RNA pull-down assay showed that POLR2B binds to PBX1 pre-mRNA. **H** The 3′RACE assay collected two different lengths of PBX1 mRNA in the 3′-UTR region, which were confirmed by Sanger sequencing. **I** Overexpression or knockdown of POLR2B changed only the length of the 3′-UTR region of PBX1 mRNA and did not alter the overall expression of PBX1 mRNA. Data are presented as mean ± SD (*n* = 3 in each group). **P < 0.01 compared with the POLR2B(+)NC group^,^ and ^##^*P* < 0.01 compared with the POLR2B(−)NC group. The sum of PBX1-long type and PBX1-short type was non-sense (n.s) (red) compared with the POLR2B(+)NC or POLR2B(−)NC group. **J** qRT-PCR was used to detect the half-lives of the PBX1-long type and PBX1-short type. Data are presented as mean ± SD (*n* = 3, each group). **P < 0.01 compared with the PBX1-long type group. **K** TCGA–GBM database showcased the relationship between the expression levels of YTHDC1 and PBX1. **L** Effects of YTHDC1 on PBX1 expression; the IDVs in each group were statistically analyzed. Data are presented as mean ± SD (*n* = 3, each group). **P < 0.01 compared with the YTHDC1(−)NC group and ^##^*P* < 0.01 compared with the YTHDC1(+)NC group.

To elucidate the mechanism by which POLR2B regulates the expression of NES and SOX2, we searched the National Center for Biotechnology Information (NCBI) database and did not find any APA sites for NES or SOX2. We also accessed the Human Transcription Factor (HumanTFDB, http://bioinfo.life.hust.edu.cn/HumanTFDB) database and concluded that POLR2B is not a transcription factor of NES or SOX2. However, because POLR2B, a component of RNA Pol II, may co-regulate the expression of downstream genes with transcription factors, after analyzing the HumanTFDB, we intersected the top 30 transcription factors with the highest scores for binding to the NES and SOX2 promoter regions to obtain four transcription factors (FOXA2, ETV4, KLF1, and PBX1) (Supplementary Table 2, Fig. 6A). We evaluated the relationships between POLR2B expression and these four transcription factors using TCGA–GBM database; only the PBX1 expression level is correlated with the POLR2B expression level and has statistical significance (Fig. 6B). We also found that, among the four transcription factors, only PBX1 has APA sites, which may be regulated by POLR2B. We constructed a co-expression heatmap to illustrate the expression level relationship between POLR2B and FOXA2, ETV4, KLF1, and PBX1 (Fig. 6C). After POLR2B overexpression, only PBX1 expression was significantly upregulated, and an ROC curve was constructed (AUC, 0.916; Fig. 6D, E). The assay further found that, compared with that in HA, PBX1 was upregulated in GBM cells, and this upregulation was more distinct in GSCs (Fig. 6F). PBX1 knockdown inhibited NES and SOX2 expression (Fig. S6A) and inhibited the malignant biological behavior of GSCs, whereas PBX1 overexpression exerted the opposite effect (Fig. S6B–D).

This study further found that after POLR2B overexpression, PBX1 expression increased, and POLR2B knockdown inhibited PBX1 expression (Fig. S7A). RNA pull-down assays revealed that POLR2B bound to PBX1 pre-mRNA but not to PBX1 mRNA (Fig. 6G). After retrieving the PolyASite database (https://polyasite.unibas.ch) [47], multiple potential polyadenylate sites (PAS) were observed in the 3′-untranslated region (UTR) of PBX1 (Fig. S7B). Subsequently, the PBX1 mRNAs of two 3′-UTR lengths were obtained by 3′RACE assay and confirmed using Sanger sequencing (Fig. 6H). Moreover, pairs of particular primers for proximal PAS (pPAS) and distal PAS (dPAS) were designed. The qRT-PCR assay revealed that upregulation of POLR2B increased the utilization of pPAS; that is, the expressed proportion of PBX1 with short 3′-UTR increased, POLR2B knockdown was the opposite, whereas the total amount of PBX1 mRNA of both lengths remained unchanged (Fig. 6I). Additionally, the actinomycin D assay showed that the PBX1-short type was more stable than the PBX1-long type (Fig. 6J). Finally, rescue assays revealed that POLR2B knockdown and PBX1 overexpression reversed the inhibition of POLR2B knockdown on the malignant biological behavior of GSCs and reversed the inhibitory effects of POLR2B knockdown on the expression of NES and SOX2. The POLR2B overexpression and PBX1 knockdown reversed the promotion mediated by POLR2B overexpression on the malignant biological behavior of GSCs; it also reversed its promoting effect on NES and SOX2 expression (Fig. S7C–F). These results indicate that upregulated POLR2B shortens the length of the 3′-UTR region of PBX1 mRNA through APA action, increases mRNA stability, and upregulates PBX1 expression. Subsequently, we found a positive correlation between the expression of YTHDC1 and that of PBX1 by analyzing TCGA–GBM database (Fig. 6K). Overexpression of YTHDC1 in GSC-U251 and GSC-U373 can upregulate the expression of PBX1, while knocking down YTHDC1 can downregulate the expression of PBX1 (Fig. 6L). In summary, these experiments demonstrate the presence of the YTHDC1/circPOLR2B/POLR2B/PBX1 axis in GSCs.

### PBX1 transcription promotes the expression of NES, SOX2, and YTHDC1

**Fig. 7:**
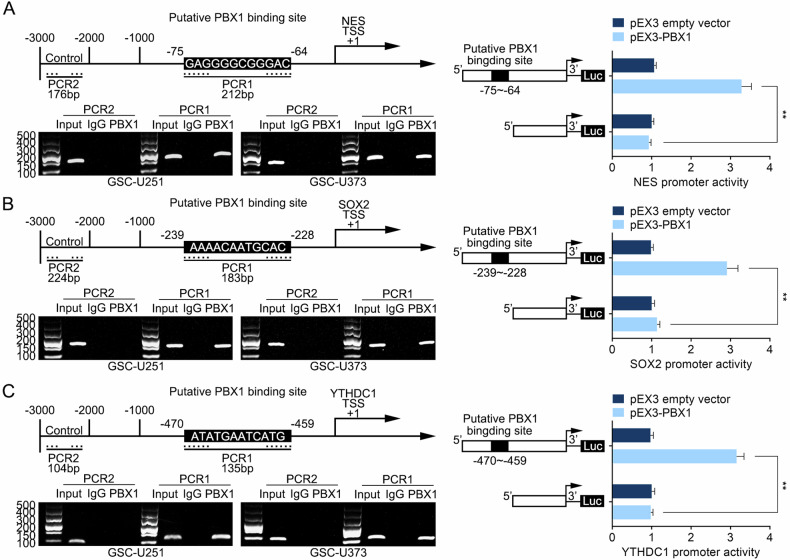
PBX1 transcription promotes the expression of NES, SOX2, and YTHDC1. **A**–**C** The ChIP assay determined that PBX1 binds to the promoter regions of NES, SOX2, and YTHDC1. The relative luciferase activity of different report vectors in 293T cells is shown. Data are presented as mean ± SD (*n* = 3, each group). **P < 0.01 compared with the pEX3 empty vector group.

TCGA–GBM database revealed a positive correlation between PBX1 and NES expression, as well as between PBX1 and SOX2 expression (Fig. S8A). PBX1 knockdown inhibited NES and SOX2 expression, whereas PBX1 overexpression upregulated it (Fig. S8B). To further explore the role of PBX1 in regulating the expression of NES and SOX2, we scanned the promoter region and predicted potential binding sites. A ChIP assay revealed that PBX1 bound to the NES promoter region at −75, and the luciferase reporter assay showed that pEX3–NES fluorescence activity was significantly increased (Fig. 7A). Similarly, PBX1 bound to the SOX2 promoter region at −239, and pEX3–SOX2 fluorescence activity was significantly increased (Fig. 7B). These results indicate that PBX1, which is highly expressed in GSCs, plays a transcription-promoting role and upregulates NES and SOX2 expression. Notably, PBX1 expression was positively correlated with YTHDC1 expression (Fig. S8C). PBX1 overexpression upregulated YTHDC1 expression, whereas PBX1 knockdown had the opposite effect (Fig. S8D). We further found that PBX1 bound to the YTHDC1 promoter region at −470, and pEX3–YTHDC1 fluorescence activity was significantly increased (Fig. 7C). Finally, we constructed a co-expression heatmap to illustrate the transcription-promoting effects of PBX1 on NES, SOX2, and YTHDC1 (Fig. S8E).

### Combined knockdown of YTHDC1, POLR2B, and PBX1 can maximally inhibit tumor growth and prolong the survival of nude mice

**Fig. 8:**
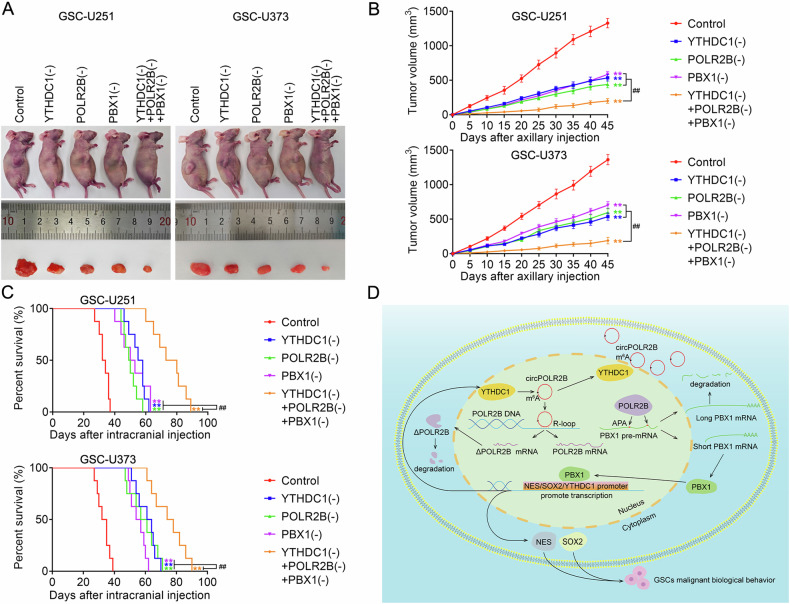
Tumor xenotransplantation and diagram of the YTHDC1/circPOLR2B/POLR2B/PBX1 pathway. **A** Tumor formation in nude mice carrying GSC-U251 and GSC-U373 cell suspensions. Tumor samples removed from each group are shown in the figure. **B** The tumor growth curve shows that after injections of GSC-U251 and GSC-U373 cell suspensions, the tumor volumes were calculated every 5 d. The tumors were removed after 45 d. Data are presented as mean ± SD (*n* = 3, each group). **P < 0.01 compared with the control group and ^##^*P* < 0.01 compared with the YTHDC1(−), POLR2B(−), or PBX1(−) groups. **C** Survival curve of nude mice with orthotopic xenografts (*n* = 8, each group). **P < 0.01 compared with the control group, ^##^*P* < 0.01 compared with the YTHDC1(−), POLR2B(−), or PBX1(−) groups. **D** Molecular mechanisms of the YTHDC1/circPOLR2B/POLR2B/PBX1 pathway in GSCs.

To determine the effects of YTHDC1, POLR2B, and PBX1 on tumor xenotransplantation, nude mice were divided into five groups. The subcutaneous xenograft tumors in the YTHDC1(−), POLR2B(−), and PBX1(−) groups were small, but the subcutaneous xenograft tumors in the YTHDC1(−) + POLR2B(−) + PBX1(−) group were the smallest (Fig. 8A, B). In the tumor orthotopic transplantation experiment, the survival analysis of the nude mice showed that the individual knockdown of YTHDC1, POLR2B, or PBX1 could prolong survival. The group with the combined knockdown of YTHDC1, POLR2B, and PBX1 presented the longest survival duration (Fig. 8C).

In conclusion, high YTHDC1 expression relieves POLR2B inhibition by altering the nucleocytoplasmic distribution of cirPOLR2B and upregulates PBX1 expression through APA action. PBX1 promotes NES, SOX2, and YTHDC1 transcription, forming a positive feedback loop comprising the YTHDC1/circPOLR2B/POLR2B/PBX1 axis to regulate the biological behavior of GSCs (Fig. 8D).

## Discussion

GBM is the most fatal intracranial tumor owing to its high invasiveness and recurrence rates [48, 49]. Accumulating evidence has shown that GSCs have a strong self-renewal ability and are the initiating cells of GBM [50, 51]. NES and SOX2 are biomarkers of GSC stemness [49, 52]. Among them, NES has been reported to have high expression that can promote the malignant biological progression of GSCs [36]. SOX2, however, is a renowned stemness maintenance factor for GSCs and promotes cell self-renewal [53, 54]. A recent study revealed that high NES and SOX2 expression is positively correlated with GBM malignancy and poor prognosis [55, 56]. Therefore, exploring the mechanisms underlying the regulation of NES and SOX2 expression and targeting GSCs has become crucial for the treatment of patients with GBM.

M6A is the most common eukaryotic RNA modification [57, 58], and m6A readers participate in regulating the growth, proliferation, and invasion of multiple tumor cells [45, 59]. In this study, YTHDC1, a protein that is upregulated in and maintains the stemness of GSCs, was screened. According to previous studies, YTHDC1 promotes the stemness maintenance of head and neck squamous cell carcinoma stem cells and promotes disease progression [60]. Similarly, YTHDC1 maintains the stemness of leukemia stem cells and accelerates their self-renewal efficiency [61]. Our study confirmed the high expression of YTHDC1 in GSCs and its ability to promote malignant biological behavior and upregulate the expression of NES and SOX2 in GSCs. An ELDA experiment also demonstrated the importance of YTHDC1 in maintaining the stemness of GSCs. The ROC curve can easily detect the ability of any boundary value to recognize diseases, and is therefore used for the diagnosis of numerous conditions [62, 63]. The ROC curve here (AUC, 0.702) suggested that YTHDC1 has a significant value in the diagnosis of GBM. Additionally, YTHDC1 is upregulated in several cancers, suggesting a cancer-promoting role of YTHDC1 [60].

Previous studies have found that circRNAs also participate in regulating GBM, colorectal cancer, bladder cancer, and other cancers and are key regulators of the tumor cell cycle, proliferation, invasion, and migration [64, 65]. In the present study, circPOLR2B expression was downregulated in the nuclei of GSCs. CircPOLR2B overexpression inhibited the malignant behavior of GSCs, suggesting that circPOLR2B may act as a tumor suppressor. qRT-PCR and FISH assays revealed that YTHDC1 downregulates the expression of circPOLR2B in the nuclei of GSCs through m6A methylation via the nuclear output effect.

As the parental gene of circPOLR2B, POLR2B is highly expressed in hepatocellular carcinoma, and high POLR2B expression is positively correlated with poor prognoses in lung cancer patients [66, 67]. POLR2B overexpression also promotes the malignant behavior of GSCs. We plotted the ROC curve of POLR2B (AUC, 0.795) and found that circPOLR2B and its parent gene formed an R-loop structure that regulates the biological behavior of GSCs. Increasing evidence has shown that tumor cells use the R-loop to regulate gene expression by affecting epigenetic regulation and transcriptional initiation and elongation [68, 69]. Meanwhile, the R-loop promotes transcription by preventing DNA methyltransferase 1 (DNMT1) from binding to DNA and by protecting the promoter region of BMP and activin membrane bound inhibitor (BAMBI) from methylation [70]. Herein, S9.6 dot blot assays revealed that the R-loop may widely exist in GSCs. DRIP and DNA–RNA FISH assays confirmed that circPOLR2B and POLR2B form an R-loop structure. The assays further revealed that circPOLR2B could bind to POLR2B in exon 15, block the transcription of RNA polymerase II (Pol II), and downregulate the expression of POLR2B. CircPOLR2B downregulation in the nucleus reduced the formation of the R-loop structure, which relieved the inhibitory effect of the R-loop on POLR2B transcription and regulated the biological behavior of GSCs. Reportedly, the R-loop structures can cause Pol II to pause, detach, and terminate transcription [71, 72], which is consistent with the conclusion drawn in our study. These results suggest that the R-loop structure may have a space-occupying effect, which can shield the binding of some proteins to DNA and participate in the regulation of the biological behavior of cancer cells. Romidepsin is a histone deacetylase (HDAC) inhibitor and anti-tumor drug approved by the Food and Drug Administration (FDA) for the treatment of cancer T-cell lymphoma (CTCL) [73, 74]. This drug can inhibit the growth of GBM in vivo [75]. According to reports, the hyperacetylation of histone lysine residues after exposure to romidepsin can trigger the formation of R-loop structures [76]. In our study, YTHDC1, which is highly expressed in GSCs, was found to mediate an increase in the ability of circPOLR2B to exit the nucleus and a decrease in the R-loop structure within the nucleus, thus promoting the malignant biological behavior of GSCs. Therefore, Romidepsin can not only serve as a drug for the treatment of GBM, but also as a potential R-loop structure agonist in GSCs to antagonize YTHDC1-mediated circPOLR2B efflux from the nucleus.

The transcription factors that may bind to the NES and SOX2 promoter regions were analyzed. PBX1 was selected as the focus of the study and the ROC curve of PBX1 was plotted (AUC, 0.916). Upregulated PBX1 expression was found to promote the malignant biological behavior of GSCs. The analysis revealed a positive correlation between the expression of POLR2B and PBX1, and multiple potential APA sites in PBX1 were noted. APA action causes the same mRNA to have different lengths of 3′-UTR tails. The shortening of 3′-UTR has been identified as a cell proliferation marker, which is significant for the self-renewal ability of stem cells [77]. PBX1 mRNAs with two 3′-UTR lengths were obtained through 3′RACE assays. PBX1 mRNA with short 3′-UTR was more stable, which may have been associated with the loss of AU-rich elements and miRNA target sites [78]. POLR2B overexpression did not result in the loss of the CDS region of PBX1 mRNA, but it did result in the APA site moving forward, the proportion of short 3′-UTR PBX1 mRNA increasing, and PBX1 protein expression increasing. PBX1 expression was further found to be positively correlated with the expression of NES, SOX2, and YTHDC1. The binding of PBX1 to the promoter region of the above genes and the transcription-promoting effects of PBX1 were verified using ChIP and luciferase reporter assays. TCRS-417 (T417) is a small molecule compound that effectively inhibits the self-renewal and proliferation of ovarian cancer stem cells by targeting PBX1 and interfering with the interaction of PBX1–DNA [31]. Similarly, T417 inhibits the proliferation of multiple myeloma cells by inhibiting the PBX1–FOXM1 axis [79]. However, the treatment of T417 in glioma has not been reported yet.

Finally, GSC-U251 and GSC-U373 xenograft assays showed that the knockdown of YTHDC1, POLR2B or PBX1 alone reduced the growth of xenograft tumors and prolonged the survival of nude mice. The mice treated with the combined knockdown of YTHDC1, POLR2B, and PBX1 survived the longest. These results demonstrate the potential clinical value of targeting YTHDC1, POLR2B, and PBX1 in treatments for GBM. Unfortunately, romidepsin and T417 as tumor targeting drugs and targeting the YTHDC1/circPOLR2B/POLR2B/PBX1 axis were not examined in the present study. The cytotoxicity of Romidepsin and T417 on GSCs and the effect of their individual or combined use on the survival of nude mice in xenograft tumors also warrant further study.

## Methods

### RNase R digestion assay

RNase R was used to detect the expression of circRNAs. In brief, 2U RNase R (Lucigen, Madison, WI, USA) was added to 1 mg of total RNA and digested at 37 °C for 10 min.

### Actinomycin D assay

Actinomycin D was used to detect the half-life of mRNA and circRNAs. DMSO (0.1%) was used to dissolve actinomycin D to a final concentration of 1 µg/mL. One milliliter of actinomycin D (Noble Ryder, Beijing, China) solution was mixed with 1 × 10^6^ cells; the cells were collected after 0, 3, 6, 9, and 12 h of treatment for qRT-PCR assays.

### RNA-sequencing (RNA-seq)

RNA was extracted from three GBM cell samples and three corresponding GSC–GBM samples using TRIzol reagent (Life Technologies Corporation, CA, USA). RNA integrity was evaluated using 1% agarose gel electrophoresis, and RNA-seq libraries were generated. Sequencing libraries were generated using the V AHTSTM mRNA-seq V2 Library Prep Kit (Illumina, CA, USA). The gene expression values of the transcripts were computed using StringTie (V1.3.3b).

### CircRNA microarray analysis

Nuclear RNA extracted from GSC cells overexpressing YTHDC1 was aligned to the reference genome using BWA (V1.5), and circRNAs were identified using CIRI2 (V2.06). CircRNA expression was calculated using the RPKM formula. DESeq2 (V1.12.4) was used to analyze differences in expression.

### qRT-PCR

The concentration and quality of the RNA were determined using a NanoDrop2000 spectrophotometer. A One-Step TB Green®PrimeScript™RT-PCR Kit (Takara, Liaoning, China) was used to detect the expression of YTHDC1 and circPOLR2B, POLR2B, PBX1, GAPDH, and U3 mRNA on an ABI 7500 Fast Real-Time PCR system. Results were obtained using the 2^−ΔΔCt^ relative quantification method. GAPDH or U3 were used as internal references. All primer information is provided in Supplementary Table 3.

### Protein extraction and western blotting assays

Radioimmunoprecipitation assay buffer (Beyotime, Shanghai, China) was used to lyse the cells on ice for 50 min, after which the cells were fragmented by ultrasound. The supernatant was centrifuged at 17,000 × *g* for 40 min. The protein concentrations were determined to ensure that the total protein loaded into each lane was 40 mg. The membranes were incubated with primary antibodies against the following proteins at 4 °C overnight: YTHDC1, POLR2B, PBX1, METTL3, and GAPDH (all from ProteinTech Group, Chicago, IL, USA). The membranes were then incubated with horseradish peroxidase-conjugated secondary antibody for 2 h at room temperature and developed using an enhanced chemiluminescence kit (Beyotime). The relative integrated densities (IDVs) were calculated using Gelpro32 software, with GAPDH as the internal reference. All antibody information is provided in Supplementary Table 4.

### Cell transfection

GenePharma (Shanghai, China) used pCMV3-C-GFPSpark and pGPU6-mCherry-Puro vectors to construct full-length sequence vectors of YTHDC1, POLR2B, and PBX1, as well as their corresponding empty vectors. The pcDNA3.1 vector was used to construct the full-length and corresponding empty circPOLR2B vectors. GenePharma used pGPU6-GFP-Neo and pGPU6-mCherry-Puro vectors to design short hairpin RNAs (shRNAs) and the corresponding empty vectors for the above genes. A stably transfected GSC cell line was then constructed with the drug corresponding to the vectors for ~4 weeks. qRT-PCR and western blotting assays were then performed to detect the transfection efficiency (Fig. S9A–E). All shRNA sequences are provided in Supplementary Table 5.

### Extreme limiting dilution analysis (ELDA)

The cells were isolated and seeded into 96-well ultra-low attachment plates (Corning, NY, USA), and 200 mL of cell culture medium was added. The cell-laying density was set at 50–400 cells/well. After 10 d of culture, if the cell sphere diameter was >100 μm, it was deemed positive (Fig. S9F). The online ELDA software can be found at http://bioinf.wehi.edu.au/software/elda/ [80].

### Immunofluorescence (IF) and fluorescence in situ hybridization (FISH) assays

The cells were permeabilized with 0.1% Triton-100 (Beyotime) for 15 min. Blocking was performed using 5% bovine serum albumin, and the membranes were incubated overnight at 37 °C with the anti-YTHDC1 antibody (14392-1-AP, ProteinTech Group) or circPOLR2B FISH probe (GenePharma). The following day, the cells were incubated with the fluorescence-conjugated secondary antibody Alexa Fluor 488 (Beyotime) or cy3 (GenePharma) for 1 h and stained with DAPI (Beyotime) for 5 min. POLR2B gDNA FISH Probe: chr4: 56,886,144–57,089,617, labeled with FITC, purchased from Future Biotech (Beijing, China).

### Mass spectrometry analysis

The detected protein was bound by the specially prepared anti-POLR2B antibody (Supplementary Table 4) and captured using magnetic beads. Sequences and sites were identified and analyzed using the National Center for Biotechnology Information protein database and Mascot Daemon.

### Chromatin immunoprecipitation (ChIP) assay

The ChIP assay was performed using a simple ChIP enzymatic chromatin IP Kit (Cell Signaling Technology, Danvers, MA, USA). According to the prediction of PBX1 binding sites in the promoter region of NES, SOX2, and YTHDC1 by the database, we designed 5′-TGGGAGTACCAGGACGTTCC-3′ and 5′-TCAGGAGGAGGAGCATTTGC-3′ as the forward and reverse primers for NES, respectively. Similarly, 5′-GCAGAGATTGGAGAAATTGGGG-3′ and 5′-CTGTAACACTCTCTCCGCCC-3′ for SOX2 and 5′-GATTACAGGTATGAGCCACTGC-3′ and 5′-ATATCTGATGATCCTGAGGAATTGCT-3′ for YTHDC1 were set as controls. All chip primer sequences are provided in Supplementary Table 6.

### Xenotransplantation of tumors in nude mice

Stably transfected cell lines were used during tumor implantation. Four-week-old athymic BALB/c nude mice were obtained from the Chinese Academy of Medical Sciences and randomly divided into five groups. Each mouse was administered a subcutaneous implantation of 1 × 10^6^ cells. The tumor volumes were measured every 5 d. The mice were sacrificed 45 d after the injections, and the tumors were isolated. Each nude mouse was transplanted intracranially with 2 × 10^5^ cells, and a Kaplan–Meier survival curve was generated for a survival analysis. The study was approved by the Laboratory Animal Management Group of China Medical University.

### Statistical analysis

Statistical analyses were performed using GraphPad Prism 8.03. Student’s *t*-tests, one-way analysis of variance (ANOVA), and two-way ANOVA were used for comparisons between groups. Results with *P* < 0.05 were considered statistically significant.

### Supplementary information

Supplementary Figures and Supplementary Methods

Supplementary Tables

Original File of Western Blot

## Data Availability

The datasets used and/or analyzed during the current study are available from the corresponding author on reasonable request.
