# Supplementary material for: M6A-methylated circPOLR2B forms an R-loop and regulates the biological behavior of glioma stem cells through positive feedback loops
Source: Cell Death Dis. 2024 Aug 1;15(8):554. doi: 10.1038/s41419-024-06946-6 (PMC11294345; doi:10.1038/s41419-024-06946-6)

Figure 1

1E

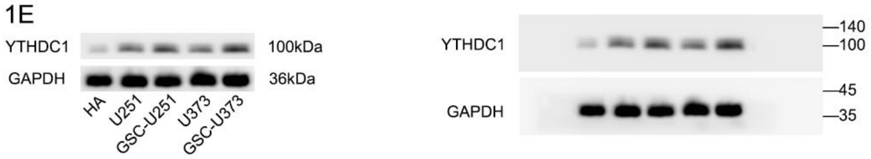

Figure 4

4B

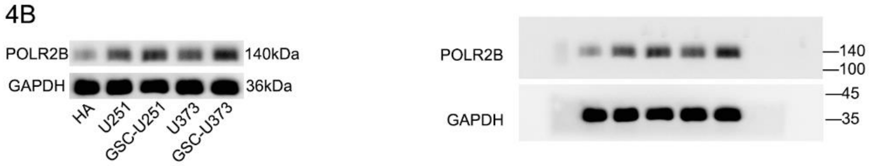

4D

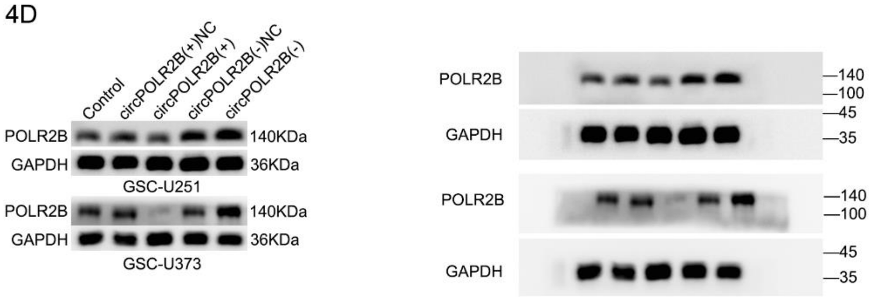

Figure 5

5G

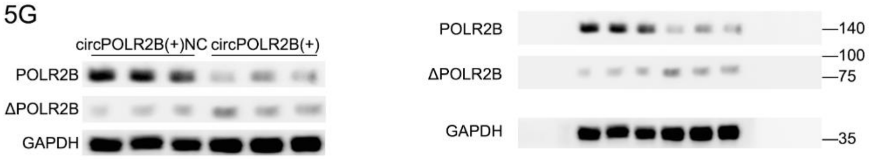

5H

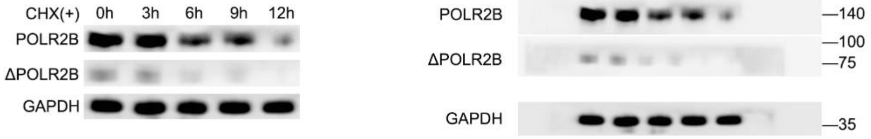

5L

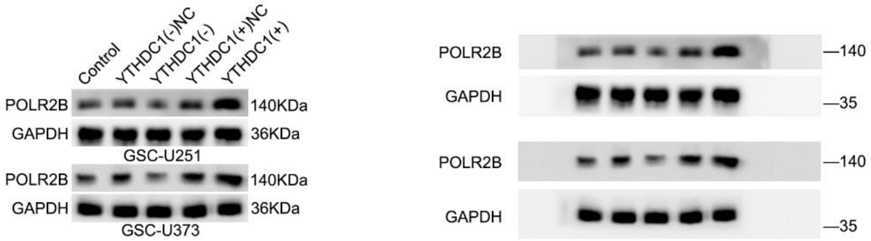

Figure 6

6L

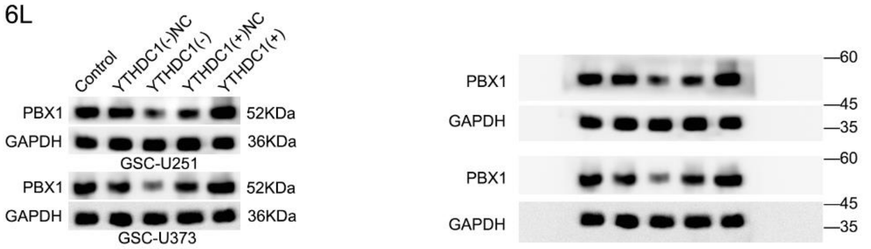

Supplementary Figure 1

S1F

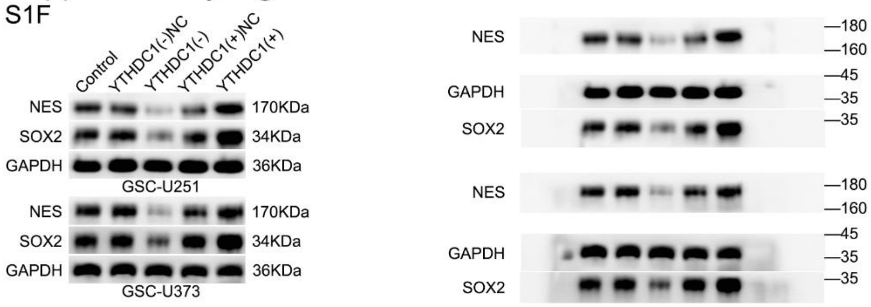

Supplementary Figure 2

S2D

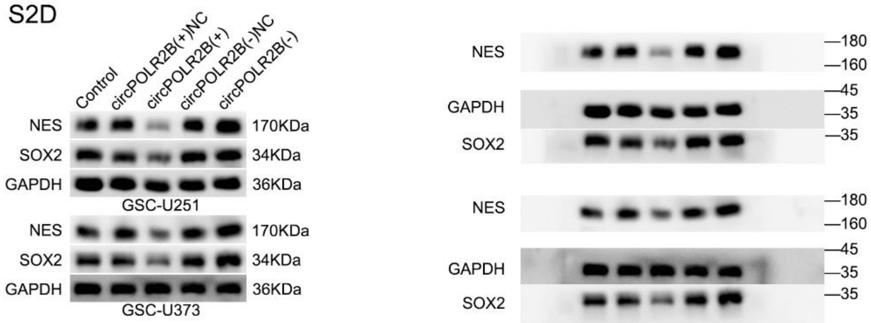

Supplementary Figure 3

S3G

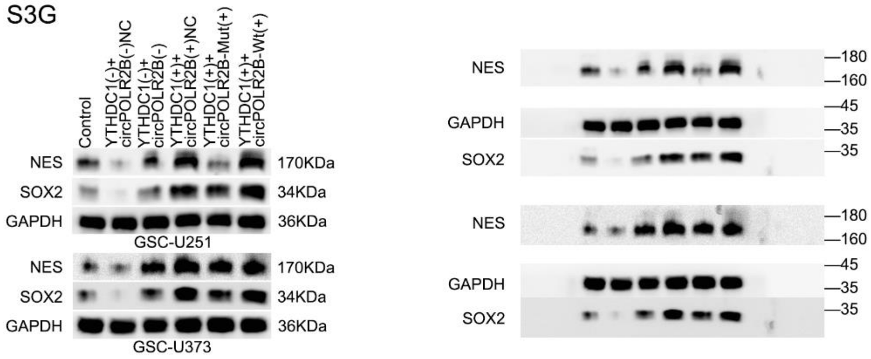

Supplementary Figure 4

S4A

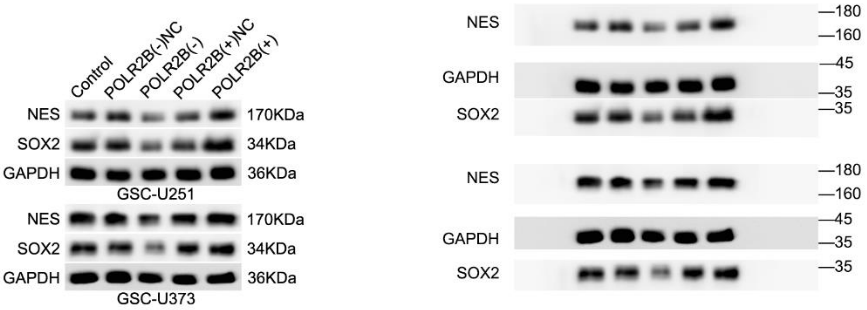

Supplementary Figure 5

S5A

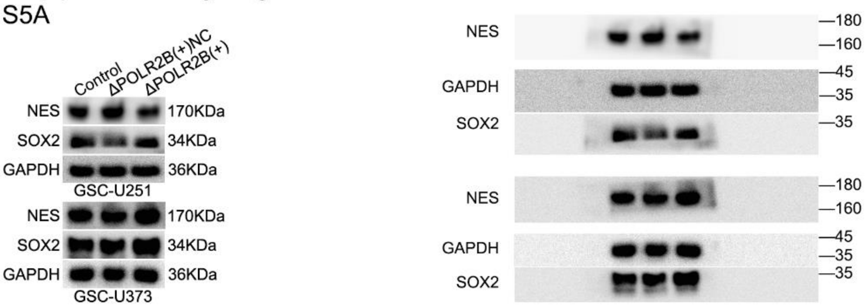

S5C

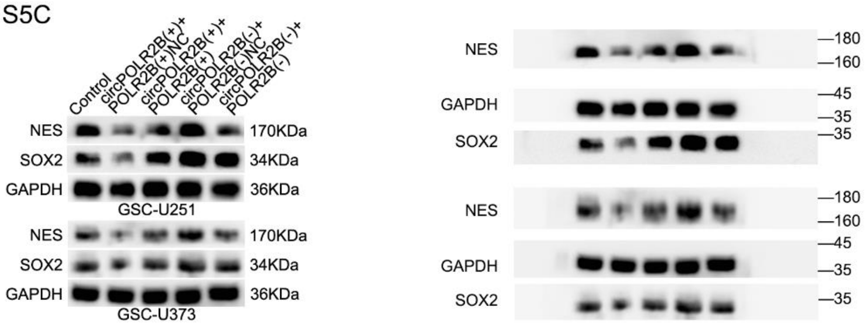

Supplementary Figure 6

S6A

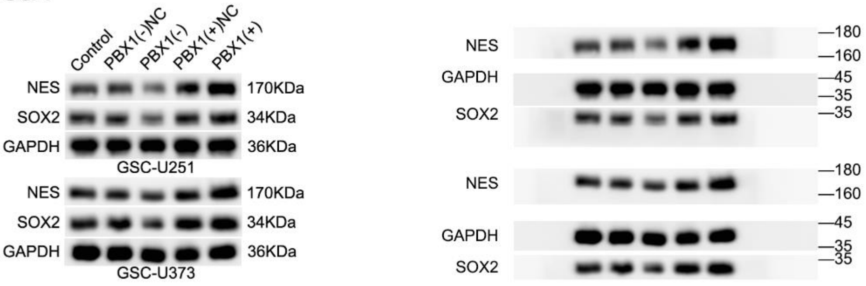

Supplementary Figure 7

S7A

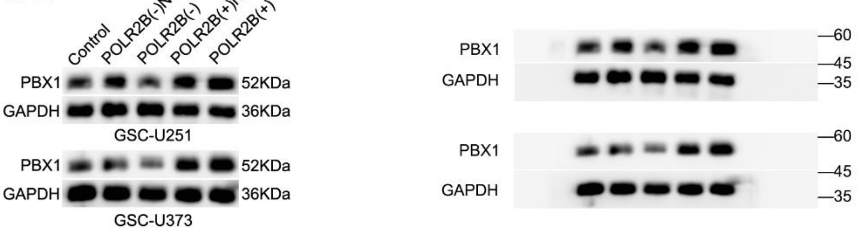

S7C

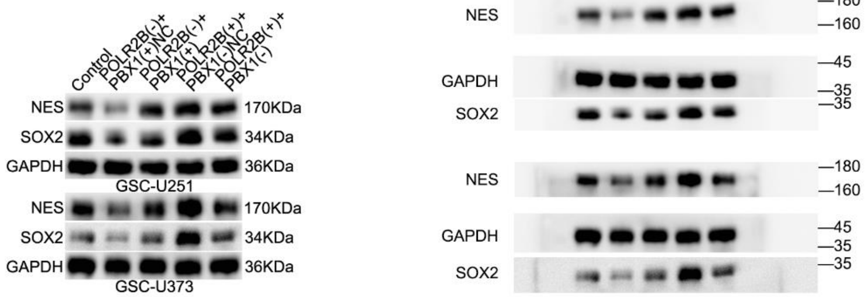

Supplementary Figure 8

S8B

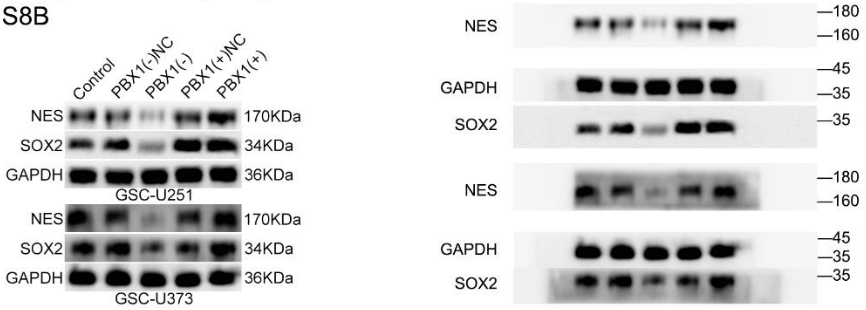

S8D

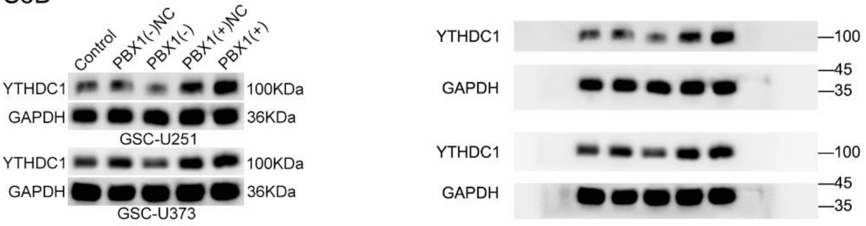

Supplementary Figure 9

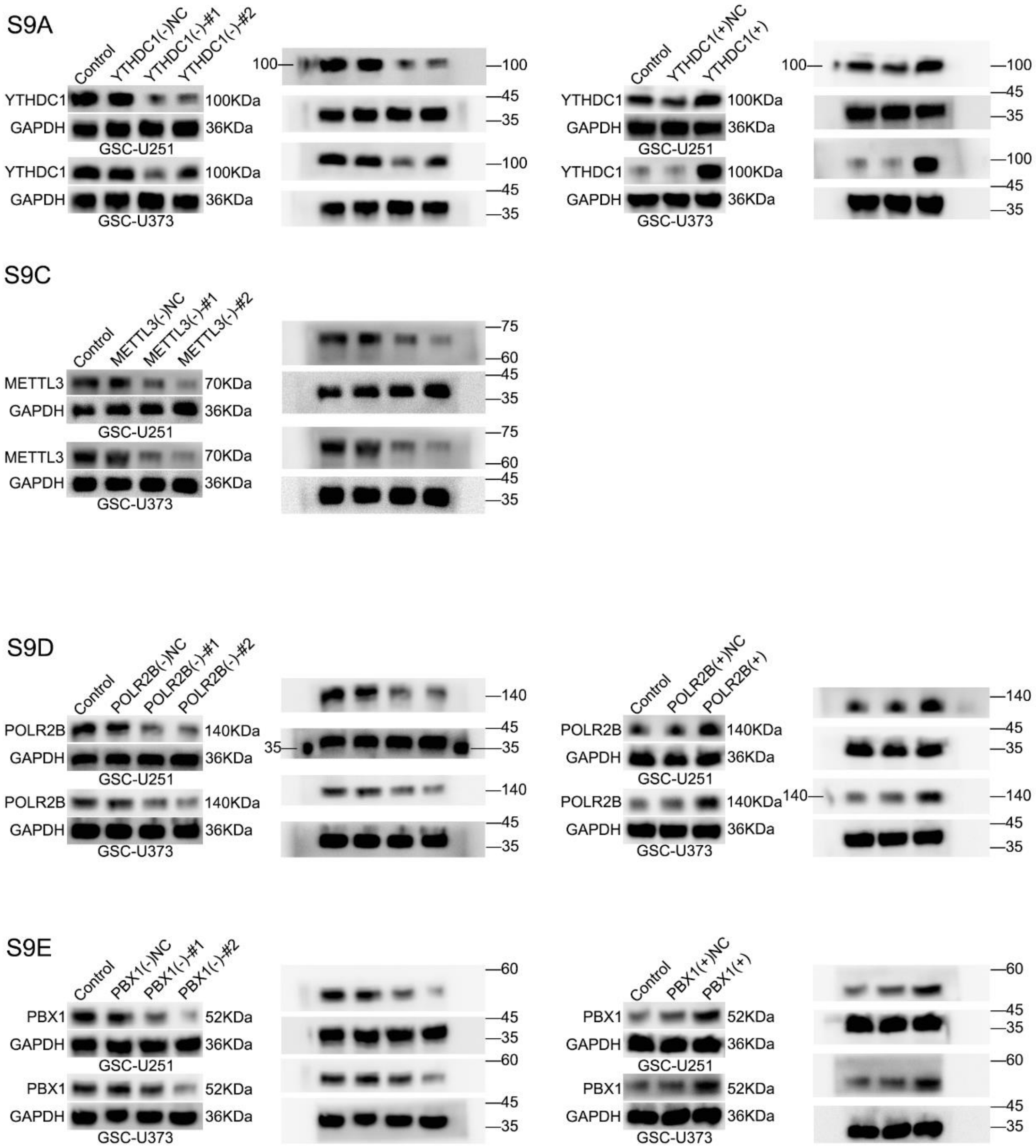

Supplement: Supplementary file 3 — Original File of Western Blot [file 41419_2024_6946_MOESM3_ESM.pdf]
