# Supplementary material for: M6A-methylated circPOLR2B forms an R-loop and regulates the biological behavior of glioma stem cells through positive feedback loops
Source: Cell Death Dis. 2024 Aug 1;15(8):554. doi: 10.1038/s41419-024-06946-6 (PMC11294345; doi:10.1038/s41419-024-06946-6)
Supplement: Supplementary file 1 — Supplementary Figures and Supplementary Methods [file 41419_2024_6946_MOESM1_ESM.docx]

**Supplementary Figures and Supplementary Methods**

**Supplementary Figure 1.** **Screening of the YTHDC1 and GBM cell lines**. (A) TCGA-database-screened genes upregulated in GBM tissue compared with those in normal brain tissue. (B) RNA-seq-screened genes upregulated in GSCs compared with those in GBM cells. (C) TCGA-database-screened gene expression relative to m6A methylation modifications. (D) The expression matrix of the YTHDC1 gene in GBM cell lines was obtained from the CCLE dataset. (E) IF showing that YTHDC1 is located within the nucleus (scale bar represents 20 µm). (F) After YTHDC1 knockdown or overexpression, the expression of NES and SOX2 in GSC-U251 and GSC-U373 was measured, and the IDVs in each group were statistically analyzed. Data are presented as mean ± SD (*n*=3, each group). ^**^*P*<0.01 compared with the YTHDC1(-)NC group and ^##^*P*<0.01 compared with the YTHDC1(+)NC group.

**Supplementary Figure 2. Screening of circPOLR2B.** (A) After the overexpression of YTHDC1, RNA was extracted from the nuclei of GSCs for circRNA microarrayanalyses. (B) CircPOLR2B was most downregulated in the nuclei of GSCs after YTHDC1 overexpression, as shown by qRT-PCR. Data are presented as mean ± SD (*n*=3, each group). ^**^*P*<0.01 compared with the YTHDC1(+)NC group. (C) qRT-PCR was used to detect the half-lives of circPOLR2B and linear POLR2B mRNA. ^**^*P*<0.01 compared with the linear POLR2B mRNA group. (D) Expression of NES and SOX2 in GSC-U251 and GSC-U373, respectively, after circPOLR2B overexpression or knockdown; the IDVs in each group were statistically analyzed. Data are presented as mean ± SD (*n*=3, each group). ^**^*P*<0.01 compared with the circPOLR2B(+)NC group and ^##^*P*<0.01 compared with the circPOLR2B(-)NC group.

**Supplementary Figure 3. M6A methylation of circPOLR2B.** (A–B) qRT-PCR and FISH assays showed that after the overexpression of YTHDC1 in GSC-U373, circPOLR2B in the nucleus transferred to the cytoplasm, while knockdown of YTHDC1 resulted in circPOLR2B remaining in the nucleus (scale represents 10 μm). Data are presented as mean ± SD (n=3, each group).^**^*P*<0.01 compared with the YTHDC1(+)NC group, and ^##^*P*<0.01 compared with the YTHDC1(-)NC group. (C) Four potential m6A methylation sites predicted by the SRAMP website and their scores. (D) Partial circPOLR2B-Mut sequence. (E) The molecular model of YTHDC1 binding to circPOLR2B. (F) After treatment with RNase R, circRNAs showed m6A methylation levels in HA, U251, GSC-U251, U373, and GSC-U373. (G) After METTL3 knockdown, the m6A methylation level of circRNAs in GSC-U251 and GSC-U373 decreased. (H) The qRT-PCR analysis found that after METTL3 knockdown, circPOLR2B retention in the nucleus increased. Data are presented as mean ± SD (*n*=3, each group). ^**^*P*<0.01 compared with the METTL3(-)NC group in GSC-U251, and ^##^*P*<0.01 compared with the METTL3(-)NC group in GSC-U373. (I) The effects of YTHDC1 and circPOLR2B-Mut or circPOLR2B-Wt on the expression of NES and SOX2; the IDVs in each group were statistically analyzed. Data are presented as mean ± SD (*n*=3, each group). ^**^*P*<0.01 compared with the YTHDC1(-)+circPOLR2B(-)NC group, ^##^*P*<0.01 compared with the YTHDC1(+)+circPOLR2B(+)NC group, ^&&^*P*<0.01 compared with the YTHDC1(+)+circPOLR2B-Mut(+) group.

**Supplementary Figure 4. Regulatory role of POLR2B in GSCs.** (A) Western blotting assay for detecting the effects of POLR2B knockdown or overexpression on the expression of NES and SOX2; the IDVs in each group were statistically analyzed. Data are presented as mean ± SD (*n*=3, each group). ^**^*P*<0.01 compared with the POLR2B(-)NC group and ^##^*P*<0.01 compared with the POLR2B(+)NC group. (B–D) The effects of POLR2B knockdown or overexpression on self-renewal (B), migration and invasion (C), and apoptosis levels (D) of GSC-U251 and GSC-U373. ^**^*P*<0.01 compared with the POLR2B(-)NC group and ^##^*P*<0.01 compared with the POLR2B(+)NC group. The scale bar represents 10 μm. (E) Schematic of the circPOLR2B-BD mutation. (F) The DRIP assay verified that circPOLR2B knockdown reduced the formation of the R-loop structure involved in circPOLR2B, whereas circPOLR2B-BD did not participate in R-loop formation.

**Supplementary Figure 5. Regulatory roles of circPOLR2B and POLR2B in GSCs.** (A–B) The expression of NES and SOX2 in GSCs after ΔPOLR2B overexpression and its impact on self-renewal efficiency. Data are presented as mean ± SD (*n*=3, each group). Non-sense (n.s) compared with the ΔPOLR2B(+)NC group. (C) The effects of circPOLR2B and POLR2B on NES and SOX2 in GSCs. Data are presented as mean ± SD (*n*=3, each group). ^**^*P*<0.01 compared with the circPOLR2B(+)+POLR2B(+)NC group and ^##^*P*<0.01 compared with the circPOLR2B(-)+POLR2B(-)NC group. (D–F) The effects of circPOLR2B and POLR2B on self-renewal (D), migration and invasion (E), and apoptosis levels (F) of GSC-U251 and GSC-U373. ^**^*P*<0.01 compared with the circPOLR2B(+)+POLR2B(+)NC group and ^##^*P*<0.01 compared with the circPOLR2B(-)+POLR2B(-)NC group. The scale bar represents 10 μm.

**Supplementary Figure 6. Regulatory role of PBX1 in GSCs.** (A) Western blotting was performed to detect the effects of PBX1 on the expression of NES and SOX2; the IDVs in each group were statistically analyzed. Data are presented as mean ± SD (*n*=3, each group). ^**^*P*<0.01 compared with the PBX1(-)NC group and ^##^*P*<0.01 compared with the PBX1(+)NC group. (B–D) The effects of PBX1 on the self-renewal (F), migration and invasion (G), and apoptosis levels (H) of GSC-U251 and GSC-U373. ^**^*P*<0.01 compared with the PBX1(-)NC group and ^##^*P*<0.01 compared with the PBX1(+)NC group. The scale bar represents 10 μm.

**Supplementary Figure 7. POLR2B and PBX1 regulate the biological behavior of GSCs.** (A) The effects of POLR2B on the expression of PBX1; the IDVs in each group were statistically analyzed. Data are presented as mean ± SD (*n*=3, each group). ^**^*P*<0.01 compared with the POLR2B(-)NC group and ^##^*P*<0.01 compared with the POLR2B(+)NC group. (B) According to the PolyASite database analysis, there were multiple potential APA sites in PBX1. The actual occurrence site is indicated in red (Figure 6H). (C) The effects of POLR2B and PBX1 on the expression of NES and SOX2 in GSCs; the IDVs in each group were statistically analyzed. Data are presented as mean ± SD (*n*=3, each group). ^**^*P*<0.01 compared with the POLR2B(-)+PBX1(+)NC group and ^##^*P*<0.01 compared with the POLR2B(+)+PBX1(-)NC group. (D–F) The effects of POLR2B and PBX1 on the self-renewal (D), migration and invasion (E), and apoptosis levels (F) of GSC-U251 and GSC-U373. ^**^*P*<0.01 compared with the POLR2B(-)+PBX1(+)NC group and ^##^*P*<0.01 compared with the POLR2B(+)+PBX1(-)NC group.

**Supplementary Figure 8. PBX1 regulates the expression of NES, SOX2, and YTHDC1.** (A) RNA-seq data of GBM were obtained from TCGA. PBX1 expression was positively correlated with NES and SOX2 expression. (B) Effects of PBX1 on the expression of NES and SOX2 in GSCs; the IDVs in each group were statistically analyzed. Data are presented as mean ± SD (*n*=3, each group). ^**^*P*<0.01 compared with the PBX1(-)NC group and ^##^*P*<0.01 compared with the PBX1(+)NC group. (C) RNA-seq data of GBM were obtained from TCGA. Expression levels of PBX1 and YTHDC1 were positively correlated. (D) Effects of PBX1 on YTHDC1 expression; the IDVs in each group were statistically analyzed. Data are presented as mean ± SD (*n*=3, each group). ^**^*P*<0.01 compared with the PBX1(-)NC group and ^##^*P*<0.01 compared with the PBX1(+)NC group. (E) Co-expression heatmap of PBX1 with NES, SOX2, and YTHDC1.

**Supplementary Figure 9. Transfection efficiency.** (A–E) Western blotting and qRT-PCR assays for detecting the transfection efficiencies of YTHDC1, METTL3, POLR2B, PBX1, and circPOLR2B; the integrated densities (IDVs) in each group were statistically analyzed. Data are presented as mean ± standard deviation (SD; *n*=3, each group). ^**^*P*<0.01 compared with the corresponding gene NC group. (F) The ELDA assay detected the self-renewal levels of GSCs, with a cell sphere diameter greater than 100 μm being considered positive. The scale represents 50 μm.

**Supplementary Methods**

***Cell culture***

Normal human astrocyte (cy-1028; HA) were obtained from ScienCell Research Laboratory (Carlsbad, CA, USA). GBM cell lines (A172, LN229, U251, and U373) and 293T cells were obtained from the Cell Resource Center of the Shanghai Institute of Biological Sciences. All cells were strictly cultured according to the American Type Culture Collection guidelines (https://www.atcc.org/). GSCs (GSC-U251 and GSC-U373) were grown using a 2% B-27 supplement (Thermo Fisher, Waltham, MA, USA), 10% epidermal growth factor (Thermo Fisher), and 10% basic fibroblast growth factor (Thermo Fisher) for more than four passages. All cells were identified using Short Tandem Repeats and Cell Line Authentication.

***Separation of nuclear and cytoplasmic fractions***

The cells were lysed using a Cytoplasmic and Nuclear RNA Purification Kit (Norgen Biotek, Thorold, Canada). The cell lysate was injected into the adsorption column and centrifuged at 6,000 rpm for 1 min to obtain cytoplasmic RNA. After the pellet had been collected and mixed with the lysate for 1 min, nuclear RNA was extracted by centrifugation at 6,000 rpm for 1 min.

***Cell migration and invasion assay***

The cell migration and invasion capacities were evaluated by observing the polycarbonate membrane (Corning) passing through a pore size of 8 μm. Matrigel (Corning) was pre laid in the invasion assay. After the addition of 10% fetal bovine serum (FBS) to the lower chamber, the cells were digested with trypsin and dispersed. FBS-free cell culture medium was added to the upper chamber, and the number of cells was maintained at 5 × 10^4^. After 48 h of culture, the cells were fixed with 4% paraformaldehyde and stained with 75% Giemsa for 3 h, and five pictures were randomly selected in each chamber under a microscope to count the number of crossing cells.

***Dot blot assay***

Total RNA was treated using RNase R, and the concentration was adjusted to 800 ng/μL. A concentration of 2 μL of RNA was dropped onto a positively charged nylon membrane (Beyotime). After UV crosslinking for 20 min, the cells were immersed in S9.6 (ab136955, Abcam, Cambridge, UK) or anti-m6A antibody (ab284130, Abcam) at a dilution ratio of 1:1000.

***Apoptosis detected by flow cytometry***

Apoptosis was detected using Annexin V-FITC/PI staining (BD Biosciences, CA, USA). After the digestion and dispersion of the cells and mixing 5 μL of PI and 5 μL of FITC under dark conditions for 15 min, the apoptotic fraction was determined using flow cytometry (FACScan, BD Biosciences). The number of cells in each group was 2 × 10^4^. We define Annexin V(-)/PI(-) as normal alive cells, Annexin V(+)/PI(-) as normal alive cells whose cell membrane is flipped due to mechanical damage, Annexin V(-)/PI(+) as early apoptotic cells, and Annexin V(+)/PI(+) as late apoptotic cells. In this study, both Annexin V(+)/PI(-) and Annexin V(+)/PI(+) were considered as apoptotic cells.

***RNA pull-down assay***

The PBX1 pre-mRNA was segmentally synthesized using a Pierce RNA 3′ End Desthiobiotinylation Kit (Thermo Fisher) and labeled with biotin. The biotinylated probe captured with streptavidin magnetic beads was then incubated with GSC lysates for 1 h at 4 °C. After elution at 37 °C for 30 min, proteins were analyzed by western blotting.

***Methylated RNA immunoprecipitation (MeRIP) assay***

Genseq and the ®m6A MeRIP Kit (Cloud-seq, Shanghai, China) were used for MeRIP detection. A concentration of 100 μL of GSC lysate set with 5 μg of anti-m6A antibody (ab208577, Abcam) was incubated overnight at 4 °C and used every other day for 40-μL magnetic bead coupling. The RNA was analyzed using immunoprecipitation and qRT-PCR.
